# Chinese ethnic dance therapy: cultural anthropology and health science perspectives on Tujia ethnic dances

**DOI:** 10.3389/fpsyg.2025.1561150

**Published:** 2025-06-06

**Authors:** Qi Mao, Wolfgang Mastnak, Ruiyuan Guan

**Affiliations:** ^1^Department of Medical Psychology, School of Health Humanities, Peking University, Beijing, China; ^2^School of Arts and Communication, Beijing Normal University, Beijing, China

**Keywords:** China, cultural sensitivity, ethnic minorities, ethno-dance therapy, Tujia dance, health promotion, World Health Organisation

## Abstract

**Introduction:**

Archaeological findings witness the anthropological roots of dance, while psychological, medical, cultural and aesthetic studies shed light on health promoting capacities and curative factors inhering in symbolic and expressive body movement. Since dance therapy became a multifaceted discipline in the middle of the 20th century, increasing evidence of beneficial effects has advocated the use of dance therapy in a broad spectrum of clinical and public health areas such as psychiatry, oncology, neurology, cardiology and geriatrics. Psychological and neurophysiological studies elucidated key mechanisms underlying dance therapeutic dynamics, and ethnological studies highlighted the wealth of indigenous dances alongside their impact on holistic well-being, hence the term ‘ethno-dance therapy’, which also relates to dance traditions of ethnic groups in China.

**Methods:**

Narrative/descriptive ethnological research provided detailed insights into dance traditions of the 55 officially recognized ethnic groups in China such as the Uyghur, Miao and Wa. Considering dance ontological perspectives, a triad of Tujia dances was chosen for this article. On this basis as well as own field studies, cultural-anthropological, psychological, physiological and neurophysiological knowledge was used to construct hypotheses about health-relevant features and factors. In terms of meta-methodology, such inferential reasoning brings about multi-disciplinary meta-syntheses, which differ considerably from the conventional understanding of this genre.

**Results:**

Our analysis of Tujia dances suggests nine distinct therapeutic principles and benefits regarding (i) cardiovascular health, (ii) musculoskeletal health, (iii) neuroplasticity and network connectivity, (iv) self-exploration and self-expression, (v) self-actualization and ontological anchoring, (vi) hypnotherapeutic dynamics and altered states of consciousness, (vii) symbolic interaction and ritualized social roles, (viii) therapeutically advantageous changes of attitudes, (ix) aesthetic immersion and the dance-self.

**Discussion:**

The broad spectrum of beneficial effects of Tujia dances may improve dance therapy in various medical areas and enhance culturally sensitive public health systems. Further research should focus on underlying mechanisms, involve dances from further ethnic groups, explore cross-cultural transferability to more precisely differentiate archetypal/anthropological and culture-dependent factors, and to clearly identify dance therapeutic functions within complex medical and psychological treatment plans.

## 1 Introduction

Since time immemorial, human cultures have given birth to a myriad of dance phenomena, and dance archaeology (Garfinkel, 2014) is about to discover anthropological sources of and reasons for symbolic movement and artistic body expression. Approaches combining comparative, developmental and cross-cultural psychology (Christensen et al., 2017) highlight the dual anthropological character of dances including aesthetic experience and neurobiological effects. Empirical evidence is given for six neural and bio-behavioral functions, i.e., attentional focus and flow, basic emotional experiences, imagery, communication, self-intimation and social cohesion.

Increasing awareness of health promoting factors and the healing potential of dance inspired systematic studies alongside distinct applications of dance in clinical areas and public health, and eventually resulted in the foundation of dance therapy as a new discipline. Arguing that dance became ‘therapeutic in the mid to late 20th century’, Kormos (2023) gave an account of the modern era of dance therapy, which focused particularly on developments in Hungary and the United States. Elucidating that the convergence of dance art and therapeutic culture engendered the development of dance-movement therapy, he refers to postmodern dance as a source of dance therapeutic methodology and lays stress on aesthetic and cultural differences between Hungary and the USA alongside their decisive impact on dance therapeutic models. This argument also applies to key principles of the present article that revolves around the complex phenomenon of Chinese ethno-dance therapy.

Regarding the international realm of dance therapy, we may distinguish between three complementary approaches dealing with (i) therapeutic features of dance traditions and genres, (ii) dance therapeutic models to treat manifest diseases or alleviate symptoms, and (iii) underlying mechanisms, e.g., psycho-affective dynamics and neurophysiological processes. By way of illustration, Expressive Flamenco (Sánchez García and Pinna-Perez, 2021) is meant to improve psycho-social, spiritual and aesthetic connections to the unconscious, as well as to help practitioners to ‘transcend the self into divine connection with their authentic self’, while more clinical or developmental psychological applications relate to topics such as trauma therapy (Koch et al., 2019) and personal growth of children with special needs and cognitive impairment (Mastnak, 2022). Regarding beneficial effects on the motor system, Tango therapy showed efficacy in improving gait speed and mitigating the decline of functional mobility (Bracco et al., 2023) alongside improvement of motor and non-motor symptoms in patients with Parkinson’s disease (Docu Axelerad et al., 2022); and dance-music-poetry integrating ‘Sega’ in Mauritius became object of therapeutic and health promoting studies involving children with special needs as well as older people with mild dementia and/or deficient social inclusion causing psycho-affective issues (Mastnak et al., 2021).

Increasing evidence of beneficial effects advocates dance therapy for a broad spectrum of medical conditions such as to improve peak oxygen consumption VO_2_ and health-related quality of life (HRQOL) in patients with chronic heart failure (Gomes Neto et al., 2014) and to modulate Parkinsonian gait alongside positive effects on balance, functional mobility and cognition (Hasan et al., 2022). While these effects mainly depend on physiological, e.g., cardiorespiratory, parameters or distinct synchronization within the brain’s motor system, dance therapy showed benefits in complex pathological and psychosomatic areas, e.g., oncology and psycho-oncology, as well. Statistically significant improvements were found in favor of community dance for ‘functional capacity, fatigue, quality-of-life and depression’ (Nelson et al., 2023), while differential oncology advocates the identification of specific dance therapeutic benefits for patients with various oncological conditions such as breast cancer (Goodill, 2018).

Despite numerous studies suggesting considerable dance therapeutic benefits for cancer patients, Cochrane reviews surprise with statements such as ‘we did not find support for an effect of dance/movement therapy on depression, stress, anxiety, fatigue and body image’ (Bradt et al., 2015). These contradictions will not harm positive results, but rather unearth the shortcomings of Cochrane paradigms and practices alongside serious inherent systemic weaknesses. Not only that they solely assess studies with reference to their own standards, while widely ignoring scientific epistemology and correspondence theory of truth, they also neglect obvious mistakes. For instance, randomization does not necessarily create analogous samples, hence the necessity that RCTs take the estimated difference between the means of an intervention group and its control into account. Science-epistemological, mathematical and statistical research is about to reveal the multiple shortcomings of several standardized evidence-oriented research models, and novel designs such as dynamic simulation or virtual cohort studies are expected to replace what is sometimes called the ‘gold standard’ in evidence- based research. This innovation also applies to dance therapy and will provide complex dynamic models that combine quantitative and qualitative parameters, and respect both individualized medicine and cultural sensitivity.

Evidence-oriented research is designed to evaluate effect-sizes but cannot explore the nature of healing processes. Comparing the back-box of (early) behavioral psychology with principles of evidence based medicine, i.e., ‘not to look into,’ the cognitive revolution with its ambition to discover internal mental processes driving human behavior resembles today’s interdisciplinary attempts to discover profound curative principles. Advanced ethno-therapeutic research integrates psychological thought, e.g., about cognitive processing and depth-psychological symbolization, neuroscientific techniques such as functional magnetic resonance imaging, and health-related wisdom inhering in cultural traditions such as communication with spirits in Mongolian shamanism.

Being crucial for developmental, re-constructive and rehabilitative cortical changes, neuroplasticity is a key factor of dance therapy. In this context, a systematic review from Brazil (Teixeira-Machado et al., 2019) summarizes that dancing is able to alter brain volumes and structures, brain functions, psychomotor adjustment, as well as levels of neurotrophic factor. All included studies demonstrated positive structural or functional changes relating to increased hippocampal volume, grey matter volume in the left precentral and parahippocampal gyrus and white matter integrity. Functional changes included alterations in cognitive performance such as significant improvement in memory, attention, body balance and psychosocial parameters. Further studies on dance and neuroplasticity coincide with these findings and highlight dance-related improvement of functional connectivity, cognitive performance and increased brain volumes in healthy older people (Nascimento, 2021), as well as positive effects on cognitive deterioration in older adults with mild cognitive impairment (Wu et al., 2021).

In addition to these genuine neurophysiological benefits, a recent systematic review (Foster Vander Elst et al., 2023) emphasized dynamic interconnections between music and dance, as well as engaged overlapping brain networks involved in perception, action, emotion and–of particular importance–eudaimonia, hence the used term ‘hedonic brain networks’. Addressing the complexity of neurophysiology, psycho-affective dynamics and cultural factors, such studies hit a key issue of our article, i.e., the complementarity of culturally sensitive factors of dance therapy and anthropological invariant conditions. In other words, while dance movement in general will impact on neuroplasticity, regardless of its cultural appearance, the artistic features of dance are brimming with meaningful symbols, biographical significance and cultural familiarity. These facts inspired the authors’ ‘three spheres model’ of dance therapy, the inner sphere representing genetically determined and anthropologically (relatively) invariant neurophysiological conditions, the middle sphere describing psychological mechanisms such as cognitive or subconscious processing, and the outer sphere consisting of distinct cultural phenomena.

While the inner sphere is inextricably intertwined with genetic, epigenetic and neuroscientific conditions, the middle and particularly the outer sphere embody genuine cultural features such as shape and meaning of the Baishou Dance (Li, 2020) – 摆手舞, literally ‘hand-waving dance’–of the Tujia people in Xiangxi. The dance originates from an ancestor worship ceremony and looks back over a tradition of about 1,000 years. Akin to today’s community dance therapy movements, the Sanam (Mackerras, 1985) with its gradual acceleration and characteristic movements of the neck, elbows, fingers and eyes is typical for the Uyghur people of the autonomous region of Xinjiang. Being linked to social ceremonies, this dance may evoke feelings of community-based well-being alongside ethnic identity and inclusion.

The Chinese dance genre Yangge–秧歌, literally ‘Rice Sprout Song’–(Du, 2017) encompasses rich aestheticization of daily life, which goes hand in hand with self-actualization and work-identity. Artistic modes of coping and creative transformation of issues remind of expressive catharsis and cathartic symbolization in Western dance therapy. Comparable to exorcism and healing rituals of the Shang Dynasty, Nuo dance 傩舞 (Yang, 2018), Nuo sacrifice 傩祭 and Nuo ceremony 傩仪 are meant to drive away evil influences and disease, while Dunhuang dance draws inspiration from the Dunhuang grotto frescoes in Gansu province, combining aesthetic dynamics of these images with modern dance elements. Particularly the (painted) movements of flying apsaras 飞天 Fēitiān – celestial beings that accompany Buddhas – gave rise to dance therapeutic considerations for treating obsessive compulsive disorders (mental liberation), schizophrenia and psychotic disorders, where hallucinations undergo a transformation from pathological entities to a unique source of creative art.

Concluding the impact of this introduction on the present research, we integrated (Western) view and theories of dance therapy, cultural anthropological considerations and psychological as well as medical perspectives to explore Chinese ethno-dance therapy, particularly a triad of Tujia dances, which form the main part of our article.

## 2 Methods

Broadly speaking, there is hypotheses generating and hypotheses assessing research. Evidence based medicine is closely connected with hypotheses assessing research such as randomized controlled trials and meta-syntheses. While these methods are not geared for genuine innovative research, the world of science needs substantial progress. And yet, findings that are processed in meta-analyses may converge in a hypothesis-generating way, which gave rise to new dual study designs such as ‘Neuroscience and Dance’ (Foster Vander Elst et al., 2023), which comprise both a ‘conceptual framework’ and a ‘systematic review’.

While systematic reviews and meta-syntheses follow operationalized algorithms, which enable artificial intelligence to go autonomously through relevant procedures, genuine innovative research is per se not standardized and requires human inventiveness, comprehensive thinking and epistemological talent. In other words, while in the near future AI-systems are expected to conduct systematic reviews and meta-analyses by themselves, progress in scientific research will also benefit from systemic meta-syntheses (Mastnak, 2021), which differ considerably from meta-syntheses that synthesize qualitative literature, e.g., in psychiatry (Lachal et al., 2017).

Systemic meta-syntheses involve essentially cross-disciplinary investigations, inferential reasoning and screening of relevant system-compatibility, such as in Chinese arts-based psycho-oncology (Mastnak and Mao, 2021). This model inspired the present study, which integrates data and findings about traditional dances of the Tujia population that stem both from ethnological research and own field work.

Concerning ethno-dance therapy in general and Chinese ethnic dances in particular, the second author of the present study (Mastnak, 2024) suggested a framework comprising (i) ontological identity such as determined by myths about the universe, origin of life or righteousness, (ii) social inclusion and collective support, (iii) aestheticization and expressive catharsis, (iv) symbolic exorcism, (v) trance, and (vi) mindfulness. This framework defined the first screening, which eventually resulted in the selection of a triad of Tujia dances that have, from a meta-therapeutic perspective, a complementary ‘inner logic’.

Our dance-therapeutic synthesis tried to find a/the rationale behind myths and traditional wisdom, as well as explanations given by authentic experts who stem from the ethnic culture referred to. Relating to the visible shape of dances, we referred to movement analysis systems such as the classical Laban model (Bernardet et al., 2019), which focus particularly on body dynamics, movement shapes and the dynamic relationship between body and space. A further dimension involves views from dance therapeutic schools and theories, while a last perspective is dedicated to cultural psychological and symbol-theoretical considerations. Integration of these perspectives should help to unearth the essence and inner nature of the holistic phenomenon of Tujia ethno-dance therapy.

While replication of studies are considered a standard to re-assess effects of interventions under comparable clinical conditions, regarding the present research characteristics replication could result in different and/or complementary theoretical frameworks and categetorizations of health promoting benefits of Tujia ethnic dances. Starting conditions of such a study would be identical, namely based on (i) ethnological data about Tujia ethnic dances comprising both genuine raw data and scientific analyses and considerations, (ii) relevant therapeutic, medical-anthropological and dance therapeutic theories, and (iii) complex interdisciplinary methods and meta-methodological as well as epistemological reasoning. Processing would lead to identification of health promoting and therapeutic factors and mechanisms, consequently give rise to comparative research and eventually initiate the construction of a new meta-synthetic theoretical model.

## 3 Results

On the basis of our investigations we assume that Chinese dance therapy covers three areas: (i) dance therapy related to Traditional Chinese Medicine TCM. Concerning the term TCM, we point out that the Chinese expression is 中医 or 中医学, which means ‘Chinese medicine’ or ‘Chinese medical knowledge’; given that there is only one realm of Chinese medicine, experts criticize that the use of the word ‘traditional’ is tautological; (ii) a broad spectrum of new Chinese models integrating dance in therapeutic or health promoting frameworks, as well as other relevant concepts such as rhythmic education; (iii) curative features in dance traditions of ethnic groups in China, the so called ‘ethnic minorities’. Although the third research perspective is quite new it seems to be immensely promising and may greatly impact on the future of ethno-dance therapy. This is the reason, why our findings relate to this area, particularly the Tujia ethnicity. Moreover, the Chinese realm of healing dances and dance therapy also encompasses several Western models such as Dance Movement Therapy.

### 3.1 Ethnicities in China and the culture of the Tujia people

Different from Western countries, Chinese passports also contain the ethnic background of the passport holder. About 90% of people with Chinese citizenship are Han, the other 10% belong to the 55 (officially recognized) ethnic minorities, which importantly contribute to the cultural richness in China. Protection of their traditions and support of their welfare is a major aim of Chinese policies, and concerns their language and culture, equal rights and enhanced self-organization.

A wealth of ethnic minorities witness the complex and dynamic history of East Asia, such as the Hmong–in Chinese language 苗族 Miáozú–in southwest China as well as in Vietnam, Laos and several other countries, or the Uighur–in Chinese 维吾尔族 Wéiwúěrzú–in the Xinjiang province in the northwest of China. Moreover, there are Korean ethnic groups, the 朝鲜族 Cháoxiǎnzú, in the northeast of China, a region that has been importantly influenced by Russian cultures. Some ethnic groups look back over very long traditions, even comparable to that of the Han. One of them is the Tujia population, 土家族 Tǔjiāzú, who lives in the mountains of the Hunan province. This region has become particularly famous as the site of the movie ‘Avatar’. As far as we know, all these cultures contain healing dances and dance rituals, often combined with music and dramatic elements, which can be understood as ethnic dance therapy. Further interdisciplinary anthropological, ethno-medical and dance-therapeutic research is needed to unearth the richness of these traditions, as well as their relevance for culturally sensitive public health systems.

The Tujia ethnic group, in Tujia language ‘Bifzivkar’, inhabits the mountain regions at the border between central and western China, i.e., the provinces Hunan, Hubei and Chongqing. Exploring the origins of Chinese dance therapy, we came across with roots dating back to the time of the Tujia settlement in today’s Zhangjiajie 张家界 region. The Tujia core area comprises the Wuling Mountains 武陵山脉 with abundant rainfall and high humidity as climatic characteristics (which is for researchers sometimes tough to bear). The unique environmental and climatic conditions had a distinct impact on how the Tujia formed their living spaces and cultures. Villages were (and still are) surrounded by magnificent mountains, steep canyons and quickly moving clouds, which evoked their ancestors’ mystical fantasies and eventually gave rise to their dance symbolism. Rituals to harmonize the coexistence of humans and celestial beings used body language, and dance-dramatic performances should make the spirits benevolent. A wealth of utensils was created to strengthen religious memory, and primitive mysterious Tujia dance became one of the hallmarks of their spiritual culture.

Comparable with myriads of archaic and animistic cultures on the blue planet, also the Tujia were highly influenced by environmental factors and tried to control the ‘spirits behind’. Such similarities suggest common anthropological roots and go hand in hand with the inner sphere of dance therapy as described above. For instance, the Tujia people worship the white tiger as their ancestor. Symbolically transformed, the animal’s rugged, heroic and vigorous nature inheres in many ritual dance movements of the Tujia and characterize a lot of folk dances as well. In a similar way, traditional Tujia dances express, for instance, how animals wave their tails, walk, hunt or wash their faces. In sum, this holistic body-soul and human-spirit balance, which importantly embodies health philosophies, has made Tujia dances a valuable cultural heritage in China.

### 3.2 The Tujia dance triad and the holistic balance of health

The result section of this article revolves around the dance therapeutic features of a distinct triad of traditional Tujia dances, (i) the Hand Waving Dance 摆手舞 Bǎishǒuwǔ, which relates to work, vitality and the spices of life, (ii) the Bronze Bell Dance 铜铃舞 Tónɡlínɡwǔ, which is a key ritual to worship nature and the ancestors, and (iii) the Maogusi Dance 毛古斯舞 Máogǔsīwǔ, which is a spiritual means to communicate with ghosts, including offering sacrifices and prayers for the future.

This triad may (or should) be understood as representation of a holistic health system, which is based on the ontological balance between three pillars of existence: (i) the actual experience of being and vigorous self-actualization in life, (ii) the people’s evolutionary chain including grateful awareness of the ancestors, who handed down life alongside the wealth of culture and wisdom, and (iii) the existential forces behind, in other words, the world of spirits and gods.

In this context, health is determined by the inner dynamic equilibrium of this system, while falling out of it entails the emergence of illness. Dance is a symbolic and embodied means to celebrate, maintain and continuously readjust this ontological equilibrium, which fundamentally differs from one-dimensional principles of causality in the sense of ‘one specific remedy cures one specific symptom’.

The concept of ‘Tujia ethnic dance therapy’, characterized by cultural specificity and therapeutic value not only aligns with contemporary societal demands for physical-mental wellness but also facilitates the preservation and development of traditional Chinese Tujia culture (Rodsaaad et al., 2023; Jain and Brown, 2001). As a crystallization of Tujia wisdom, this dance form embodies profound cultural connotations intrinsically linked to ethnic traditions, customs and collective spirituality (Vermes, 2019). Through its choreographic elements—including body movements, gestures, facial expressions, costumes, and musical accompaniment—it conveys specific meanings and emotions that reflect interpretations of nature, daily life, and spiritual beliefs (Fausto et al., 2024; Aithal et al., 2023). These cultural components foster cultural identity and belongingness, thereby enhancing psychological resilience and mitigating psychological stress (Mandlik, 2015; Brown and Cameirao, 2023).

Dance inherently possesses psychotherapeutic potential through multiple pathways influencing mental health. First, it serves as an affective medium enabling emotional expression and regulation, particularly for complex emotions like grief and anger (Handayani, 2021). Second, dance enhances cognitive functions such as memory and attention through the neurocognitive engagement required for movement memorization (Su, 2024). Third, collective dance participation promotes social interaction, strengthens interpersonal relationships, and reduces loneliness and social isolation (Wildeman et al., 2024). Additionally, mastery of dance techniques and successful performances can elevate self-esteem and confidence (Cheng et al., 2024). Emerging evidence further suggests that dance improves physical functioning in older adults by enhancing balance, coordination, flexibility and fall prevention (Heiberger et al., 2011). These multidimensional benefits—spanning physiological, psychological, social, and neurological domains—collectively contribute to holistic well-being.

As cultural heritage, Tujia ethnic dance warrants deeper exploration for its mental health applications. Culturally embedded dance practices provide therapeutic interventions with contextualized significance, particularly as they embody historical narratives, value systems and lifestyle patterns of specific ethnic groups (Sun and Sun, 2009). Cultural elements such as traditional music, attire and symbolic movements can evoke ethnic identity and pride, reinforcing psychological belongingness and cultural confidence (Kim and Kim, 2013; Tang, 2023), which are critical factors for maintaining mental health amid sociocultural transitions (Mastnak, 2024; Capello, 2007; Pangestu, 2021). Moreover, culturally specific rhythms and movements may correlate with particular emotional states, facilitating affective expression and catharsis (Conner et al., 2020; Delattre et al., 2024). Group-based dance activities further enhance social cohesion and mitigate isolation through communal participation (Herdiani and Munggaran, 2023; Darko, 2024; Qu et al., 2023).

From a dance therapy perspective, the uniqueness of Tujia dance lies in its potential alignment between movement patterns and psychological states. As a nonverbal psychotherapeutic modality, dance therapy utilizes bodily expression for emotional release, stress reduction and self-awareness enhancement (Auliya and Yudiarso, 2022; Cai et al., 2023). Symbolic movements in Tujia dance such as nature mimicry, emotional storytelling, or harvest celebrations may aid in emotional articulation (Agung Prameswari, 2021; Fahrurrozi and Yuda, 2022). The accompanying music and rhythms also exert mood-modulating effects, with lively tempos energizing participants and slower melodies inducing relaxation (Ning, 2023; Zhao et al., 2022). Thus, integrating Tujia dance into therapeutic frameworks offers culturally sensitive and creative mental health interventions.

Empirical studies highlight the Hand-Waving Dance 摆手舞, a quintessential Tujia cultural symbol, as more than mere entertainment; it serves vital sociocultural functions in festivals, rituals and communal prayers (Tang, 2023). Participation strengthens ethnic identity and psychological well-being, particularly crucial during rapid industrialization that threatens traditional lifestyles (Zafeiroudi, 2023). Academic investigations into its primordial and modern functions aim to optimize its role in societal harmony and comprehensive well-being (Zu-guo and Wan-hong, 2010). The dance’s pedagogical structure maximizes learner autonomy while fostering collective consciousness and aesthetic harmony through unique relational dynamics.

Consequently, developing Tujia dance therapy represents a necessary synthesis of cultural preservation and mental health imperatives. By harnessing its cultural and therapeutic values, this approach can advance individual wellness while safeguarding intangible heritage. The question ‘What does Tujia dance therapy heal?’ must be posed in a way that complies with the Tujia’s systemic view of health.

From a meta-theoretical point of view, the contradiction between quantitative evidence-based medicine (in the sense of Cochrane principles) and complexity science in systemic health care (Chandler and Hopewell, 2013; Joachim and Carmel, 2013) springs to mind. While conventional evidence-based medicine narrows its epistemological value down to effect sizes (which are, from a mathematical and medicine theoretical point of view doubtable too), complexity science in medicine advocates a dynamic multi-factorial correspondence theoretical approach. From this perspective, the Tujia dance triad that we are dealing with becomes surprisingly modern and resembles much more the paradigms and features of complexity clinical and public health science than monocausal therapeutic approaches.

Moreover, we find a myriad of similarities between the Tujia dance triad and Western therapeutic approaches and schools of thought. The Hand Wave Dance is akin to cognitive behavioral approaches which support positive attitudes towards one’s actual existence alongside embodied self-actualization, vigorous self-identity and the important role one plays for fruitful social dynamics. The Bronze Bell Dance goes hand in hand with the awareness of being part of the nature as well as the genetic and epigenetic transmissions that determine our deep biological determination as well as cross-generational experiences. These processes involve mindful self-discovery and complement existential philosophical issues in counselling, psychotherapy and psychiatry. And while many conventional Western methods work with cognitive reasoning and verbal exchange, the Tujia dance triad uses symbolic experience and an embodied ‘recognition’ with high health promoting impact.

From a dance theoretical and dance therapeutic point of view, these three dance entities are strikingly different. While the Hand Waving Dance may remind of social dances, the Bronze Bell Dance can be regarded as a dance-dramatic ritual, and the Maoguisi Dance contains Shamanic features. These differences also impact on their socio-cultural function and embedment. Together with dance therapeutic analyses, the following sections present and discuss findings about this Tujia dance triad.

### 3.3 Positive vitality: the hand waving dance

Different to numerous ethnological studies and documentary data about the Hand Waving Dance, which is at the same time the most popular one of this triad, there is, to our knowledge, still a complete lack of research on its therapeutic and health promoting features.

How practitioners of this dance described its features and impact on individuals as well as the entire society, served as initial source of our health-oriented considerations. They emphasized that the dance symbolizes, similar to work-dances in other cultural traditions, both features of and identity with labor, which also explains its numerous variations.

However, a deeper look at its characteristics as well as the dancers’ attitudes and experiences reveals that the Hand Waving Dance goes far beyond mere transpositions of labor into artistic media, and our analyses suggest seven factors with relevance to therapy and health care: (i) consistent identity and self-actualization, (ii) contribution to and celebration of perpetual development, (iii) embodied vigor and holistic energy, (iv) joy of being and hedonic sensitivity, (v) social inclusion and mutual emotional security, (vi) mindful liberation and bodily flow, and (vii) holistic (ontological) synchronization.

While originally self-actualization was at the top of Abraham Maslow’s hierarchy of needs, meaning that it rarely could be achieved, several psychological positions have underscored the crucial importance of giving full play to one’s creative, intellectual and social potential, which is confronted with the dual challenge of tackling life conditions and adjusting attitudes. Practitioners may experience the Hand Waving Dance as a means to consolidate self-acceptance and role-identities in a way that also allows profound changes following mindful self-liberation (see point vi).

The wide and rotating arm movements of the Hand Waving Dance call the idea of perpetual dynamics of existence to mind, although the Buddhist belief in reincarnation and the Daoist concept of eternal transformation do not belong to genuine Tujia thought. Concerning the dynamic dance shapes, we recognized considerable differences, though. While highly artistic performances were dominated by controlled arm movements, more common performances often tended to make use of biophysical impulse-interactions, which reminded somehow of warming-up exercises in sports. But they expressed a strong innate impetus, why we assume that the second mode has higher identity characteristics than the ‘professional’ one. This observation also relates to mindful liberation and bodily flow. We recognized a highly natural use of biophysical responsiveness, a substantial interaction between body-awareness, muscular action and the laws of inertia and gravitation. Regarding hypotheses alongside unsolved issues, we intend further studies on this phenomenon using electrophysiological and qualitative sensory approaches.

This dissimilarity between celebrating (cultural authenticity) and performing (artistic genre) the Hand Waving Dance also affects what we refer to as embodied vigor and holistic energy. Experienced coalescence of the dance and the self seems to release energy while dissolving inner blocks. Although in Western medical and biopsychological circles the notion of energy is discussed in a very heterogeneous way, our assumption is in a sense compatible with Mihaly Csikszentmihalyi’s concept of flow, certain neuropsychological positions about dynamic network connectivity and TCM views of dynamic energetic balance. Phenomenologically, practitioners describe a positive, both psychological and physiological pervasive energy. Discussing cross-cultural transferability, we assume that this very identity with movement importantly influences the dopaminergic reward system and integrating central nervous structures such as the anterior insular cortex, which also plays a crucial role in neuroaesthetics. At the moment, these considerations have hypothetic character and require psycho-physiological studies. According to subjective experiences, this positive holistic energy goes hand in hand with a most immersive joy of being, ecstatic excitement, and releases afresh the sense of mindful self-liberation.

The Hand Waving Dance is very popular among Tujia people and performed by both common people and professional dancers. It can be regarded a group or community dance with several variations including circular shapes, formations with dispersed dancers, or dramatic changes and encounters. While in our opinion many non-professional dancers looked more authentic, both realizations express a high degree of social inclusion and community-based emotional security. Symbolization of mutual support and non-manipulative participation becomes transparent and mirrors a key human desire that is well known from family and partner therapy and expressed in countless poems: mindful inner connection together with the awareness that ‘no tree grows in the shadow of the other’. In this regard, we find a fascinating mode of dance synchronization which most obviously goes beyond pure technical co-ordination. At a certain point–particularly in non-professional Hand Wave Dance activities–we got the impression to witness the emergence of a holistic ontological synchronization and its soothing inner force.

The shape of the dance itself is characterized by typical arm swinging and rotating, often together with spiral torsions, which call Moshe Feldenkrais’s assumption about archaic principles of human body movements to mind, although dynamic features may differ. While the flowing movements of some artistic performances look elegant, other styles involve abrupt elements that remind, for instance, of the Bakse dance and its therapeutic implications. The Hand-Waving Dance, as its nomenclature suggests, primarily features distinctive hand movements that form the core of this traditional practice. These oscillatory motions simulate various productive labor activities, domestic scenarios, and combat situations through differentiated swinging patterns (Yang et al., 2018; Liu et al., 2017). The coordinated interaction between upper and lower body movements generates unique choreographic vocabulary, characterized by isotropic extensions throughout the kinetic chain (Cai et al., 2023). A fundamental movement pattern, the ‘Driving the Pig Step 赶猪步’, exemplifies this through its simulation of livestock herding. This ipsilateral limb coordination (homolateral arm-leg movement) combines knee flexion, vibratory motions and downward weight shifts. Dancers lean forward while executing alternating arm swings that mimic animal propulsion, synchronized with rapid footwork conveying pursuit dynamics. This movement not only symbolizes the Tujia ethnic group’s emphasis on animal husbandry but also metaphorically represents collective cooperation in agricultural productivity. The synchronized ‘same-side hand swing with corresponding footstep’ pattern embodies the rhythmic coordination inherent in communal labor practices. Another foundational movement, the ‘Grinding Tofu Step 磨豆腐步’, replicates tofu preparation through waist-centered rotations and continuous arm push-pull motions. Dancers perform spiral oscillations (‘four triple-time movements’) simulating millstone circumrotation, complemented by stable footwork emphasizing endurance and equilibrium in labor processes. This movement serves as both a cultural record of traditional dietary practices and a symbolic representation of meticulous craftsmanship, reflecting the Tujia people’s wisdom in natural resource utilization.

These movement patterns share common choreographic characteristics rooted in productive activities (Jianbing, 2020): (i) Sturdy movements with bold lines and dynamic force; (ii) Predominantly ipsilateral limb coordination (right arm with right leg, left arm with left leg); (iii) Restricted hand movement amplitude (generally below shoulder height); (iv) Natural torso undulations synchronized with limb movements, typically incorporating rhythmic waist rotations and knee flexion; (v) Evolving spatial formations (circular, serpentine configurations); (iv) Expressive facial relaxation with smiling countenance, conveying celebratory atmosphere.

These kinetic characteristics embody the Tujia’s pursuit of ‘harmonious natural philosophy’. The juxtaposition between the vigorous ‘Driving Pig Step’ and meticulous ‘Grinding Tofu Step’ reflects the Eastern philosophy of combining vigor and gentleness. Cyclical movement repetitions metaphorically signify life continuity and natural rhythms, while coordinated group patterns manifest ancestral wisdom in ecological adaptation and social cohesion. This embodied knowledge system preserves ethnic memory through corporeal semiotics, offering insights into minority cosmologies in southwest China. Contemporary dance analysis methodologies, including Laban Movement Analysis (Sutopo et al., 2019) and Systemic Functional Dance Discourse (Maiorani and Liu, 2023), enable granular decomposition of these movement units. Such approaches facilitate examination of kinetic quality, spatial utilization, and inter-movement relationships, potentially advancing applications in ethno-dance therapy.

### 3.4 Embodied symbolism: the Bronze Bell Dance

While everyone is invited to perform the Hand Waving Dance, the Bronze Bell Dance is only celebrated by chosen people, except ‘imitations’ or re-plays of the Bronze Bell Dance, e.g., in educational domains such as elementary or junior high schools. It is a traditional ritual worshiping the nature and the ancestors, which is a very common practice in China, though. Comparable to a myriad of other rituals, symbolic costumes and properties are indispensable, as well as the musical function of the drum, the gong and the Suona, a double-reed instrument similar to the Iranian Sorna and the Middle European Schalmai with its characteristic penetrating and highly expressive sound. These instruments are also akin to the ancient Greek Aulos, which was regarded a means to express and heal traumatic injuries of the soul.

The dance is characterized by forceful movements, body flexion, asymmetric arm spirals, broad stances and energetic leaps and stamping, dominated by vital impulses. In a repetitive way, music and dance are linked by intensifying accelerations, which culminate in a highly energetic standstill. The whole process is accentuated by interjections such as 哼 Hmph and 哈 Ha, which have no proper semantic meaning.

Comparing the three dances from an interdisciplinary perspective, we may differentiate awareness, evolution and function. The Swiss philosopher and anthropologist Jean Gebser (2020) suggested distinct phases of consciousness characterizing the evolution of the human mind, i.e., an archaic, a magic, a mythological, a rational and a holistic-integral one. According to his cultural-evolutionary framework, the Hand Waving Dance is the most ‘modern’ one, which focuses on the interplay between individuals and the society, as well as hedonistic attitudes and the delight of self-actualization.

The Bronze Bell Dance relates to Gebser’s mythological stage, which is dominated by rituals and myths, as well as transcendent ontological awareness. Profound dance experiences of being (part of the) nature as well as a transient manifestation of evolutionary and hereditary dynamics relate to the genetic bond with ancestors, as well as the epigenetic involvement in cross-generational cultural experiences. Thus, the dance contributes to self-awareness alongside gratefulness and re-adjustment of one’s being in a wider social and evolutionary context.

Regarding Jean Gebser’s theory, the next dance we are dealing with, the Maogusi Dance, is the most archaic one. According to Gebser, the human mind is characterized by predominant states, but does not abolish the previous ones. This means that also ‘modern people’ encompass features of archaic, magic and mythologic awareness, use them to experience oneself and the world, and can also be used for therapeutic purposes. This decisively impacts on therapeutic means, which may also be pre-rational, e.g., beyond the repertoire of cognitive behavioral therapy. In sum, dance therapeutic approaches inspired by these Tujia dances may involve different functional features, which are also mirrored by various psychotherapeutic approaches using rituals, trance or archaic imagination.

### 3.5 Spiritual ontology: the Maogusi dance

The Maogusi dance mirrors the roots of Tujia Shamanism and was originally the way to communicate with ghosts and to sacrifice to them. In many archaic and animistic cultures, shamanism is brimming with dance, music, dramatic elements and symbols alongside altered states of consciousness. Being one of the pioneers of research on shamanism, Mircea Eliade (1961) pointed out that the shaman merges spiritual and medical functions, he or she is a spiritual master, who usually gained his power through initiation rituals, and a healer, who cares for the health balance in his tribe and negotiates illnesses and curative means with responsible ghosts. This also pertains to the Maogusi Dance as key ritual of Tujia Shamanism.

However, these ancient practices underwent transformations, and today we mostly find two phenomena. One is both in Tujia circles and in the wider Chinese community regarded as epic dance-theatre. It revolves, similar to myriads of epic genres in various cultures, around the origin and spirit of the Tujia population. The art of telling, remembering and celebrating a people’s history belongs without a doubt to the most precious treasures of a culture. Strikingly contrasting, we also came across with simplification of the Maogusi Dance for touristic purposes, interludes on sport events or shows on funfairs. Regarding similar practices that eventually resulted in the loss of the original phenomenon, we emphatically advocate awareness of the roots and protection of cultural heritage, particularly if it is connected with health care.

From a dance therapeutic perspective, we may differentiate three possible transformations, which go beyond the original purposes, but still contain their spirit: (i) settings according to psychotic symptoms or schizotypal personality traits, (ii) communication with diseased people similar to psychodramatic and imaginative methods, and (iii) spaces for culturally sensitive spiritual interchanges and/or parapsychological experiences.

Creative dance and drama proved to be a feasible and promising means for people with psychotic hallucinations and schizotypal ideation (Howe, 2022; Moreno and Casson, 2004; Mørck and Stanghellini, 2023), to connect with their sensations in a positive way, and in some cases of medication resistance to even control them; or to take them as a source for creative developments, which may change their (often devastating) labelling as mere pathological phenomenon. Moreover, creative dance and/or dance drama can help to process symbolized (pathological) contents in a depth-psychological hermeneutic way.

This also applies to a broad spectrum of bereavement conditions, including those with pathogenic potential. For instance, in the context of child oncology, parents may want to continue communication with their deceased child and seek appropriate rituals to cope with their loss and create spiritual bonds (Yeh et al., 2000). Culturally sensitive or cross-culturally inspired psychotherapy may be nourished by individually adjusted rituals such as the Maogusi Dance, not least in cases where grieving brings about pseudo-hallucinatory sensations, e.g., hearing the voice of the deceased or seeing his or her shadow (Bilu and Witztum, 1993).

### 3.6 Cross-cultural dance therapeutic values

Globally, arts therapeutic discussions are highly diverse, particularly with regard to cross-cultural transferability. While several schools of thought have a certain tendency to generalization, in our opinion dance movement therapy DMT is a typical example, interdisciplinary circles rather differentiate between anthropological principles, which are relatively independent from specific cultural features, and cultural embedment which causes limited cross-cultural applicability. The authors of this article adhere to the second position, which encourages similar ethno-dance therapeutic research alongside comparative studies. Moreover, the present Tujia dance analyses raise the question of possible general dance therapeutic principles, which resulted in the following nine factors:

- Cardiovascular health. Dance therapy can be regarded as arts-based endurance training and improvement of maximal oxygen consumption (VO_2_ max). For this reason, it is used in cardiac rehabilitation as well as preventative cardiology. Depending on the patient’s attitude, artistic activities may be more attractive than pure cardio-training, facilitates integration of exercises into life-styles and enhances life-long sustainability. In this realm, the Fudan University in Shanghai opens 2025 a research center for cardiac rehabilitation and arts therapies with distinct focus on this topic. Research on cardiorespiratory health associated with Chinese ethnic dance traditions is intended.
- Musculoskeletal health. This area addresses the whole life-span and relates to complex functions of the locomotor system comprising muscles, adjacent connective tissues, bones and joints. In terms of neurosciences it concerns the control, adjustment and coordination of movement and related executive functions, as well as the different sports-medical forms of strengths and body flexibility. The dynamic richness of the Tujia dance triad may support musculoskeletal and neuro-motor vitality, e.g., in culturally sensitive geriatrics (Periyakoil, 2019).
- Neuroplasticity and network connectivity. Numerous studies elucidate that dance combined with music has high potential to activate the brain derived neurotrophic factor alongside neuroplasticity (Brattico et al., 2021; Muinos and Ballesteros, 2021; Toader et al., 2023), which are key factors in neurorehabilitation. By way of illustration, these therapeutic mechanisms, which also apply to the Tujia dance triad, are used at the department of neurorehabilitation of the 1st medical faculty of the Charles University in Prague to treat acquired brain injury ABI (Keller et al., 2020).
- Self-exploration and self-expression. While some dance-therapeutic models show a certain tendency to (probably) overestimate expression, the authors advocate a therapeutic balance between dance-based self-exploration and self-expression. These relate to different psychological modalities, but eventually complement each other. Self-exploration involves dance-symbolic self-experience, also as a hermeneutic gate to unconscious areas, and symbolic interaction, while self-expression is widely regarded as a cathartic mode of problem processing (Kaplan, 1975; Purcell, 2020).
- Self-actualization and ontological anchoring. Dance therapy can essentially complement self-exploration and self-expression with creative self-actualization, such as in people with chronic mental disorders who are faced with external restriction of their talents and inhibition of their inner potential (Kiepe et al., 2012). In qualitative proximity to inclusive drama in various countries, e.g., the ‘Theater Apropos’ in Munich, culturally sensitive and creative dance can serve as a space for arts-based self-realization (Schielicke et al., 2018). Related processes are likely to be entwined with existentialistic issues, and dance can become a way towards mindful ontological anchoring, sometimes described as rebirth of the ‘dancing self’.
- Hypno-dance therapy and altered states of consciousness. Shamanic and spiritual dances such as the whirling dervishes and complex practices following Mevlana Jelal’uddin Rumi, have greatly inspired transpersonal therapeutic models and pseudo-ethnic forms of hypno-dance therapy (Harel et al., 2021; Vicente, 2007). According to principles of hypnotherapy, mainly that the state of trance belongs to the human mental repertoire, dance therapy takes advantage of the trance-inducing potential of embodied rhythmic movement as well as dance symbolism (Njaradi, 2018). In sum, the authors of this article find that dance-related altered states of consciousness, which are very common in archaic cultures, are still underrepresented in the (Western) realm of dance therapy.
- Dance-drama and social roles. There are Chinese research activities revolving around aestheticization of psychodrama, as well as the integration of role-based therapies and the arts. Similar to several Western approaches, qualitative transitions from playing social roles via symbolic identification to modification of the social self are of crucial importance. According to the authors’ experiences, such processes may importantly benefit from artistic symbolization and symbolic interaction. For instance, the Hand Waving Dance is full of such elements and may inspire ethno-dance-based models of dance therapy for culturally sensitive application.
- Therapeutically advantageous changes of attitudes. While, for instance, cognitive behavioral psychotherapy systematically supports changes of pathological cognitive patterns, the authors observed spontaneous (although sometimes not sustainable) changes of adverse, e.g., obsessive-compulsive or negativistic attitudes in Tujia-associated dance therapy sessions. There are still questions about underlying mechanisms, which may involve high role-identity, dissociation of the pathological and the performed self, as well as enhanced possibilities to alter attitudes, e.g., through trance and the activation of default mode network dynamics (Hove et al., 2015). Further interdisciplinary research is needed and may inspire novel culturally sensitive dance therapeutic models.
- Aesthetic immersion and essence. Aesthetic and creative therapies are not better than others. But they are different. In several clinical cases, arts-based therapies offered new possibilities to patients with manifest therapy resistance and therapy frustration (Danquah, 2023). Dance and dance-drama not only create flexible symbolic spaces, they also respond to the aesthetic and creative repertoire of human beings, which is witnessed by phenomenological, empirical and neuro-aesthetics. Taking these facts together with cultural anthropological findings into account, dance is not an ephemeral leisure-time distraction, but a means that goes to the roots of the human nature. And this is where ethno-dance therapy originates: an ontological and transformative means to re-connect with one’s profound existential qualities.

## 4 Discussion

Chinese ethno-dance therapy essentially complies with guiding principles and key suggestions of the World Health Organisation (Napier et al., 2017): ‘incorporating cultural awareness into policy-making is critical to the development of adaptive, equitable and sustainable health care systems, and to making general improvements in many areas of population health and well-being’. Moreover, community-based ethno-dance goes hand in hand with WHO-suggestions to enhance self-care (World Health Organization, 2022), the WHA74.14 ‘Comprehensive mental health action plan 2013–2030’ and recent activities to boost global health promotion. It is the explicit intention of the authors to not only conduct research on Chinese ethno-dance therapy, but also to go in for dance-based improvement of health according to WHO resolutions and statements.

While medical questions such as the operational lifespan of a coronary bypass call for one-dimensional quantitative answers, dance therapeutic issues mostly involve complex dynamics, and new scientific tools alongside meta-methodologies and epistemological considerations are needed. Regarding medicine and public health areas, Sturmberg and Martin (2022) call complexity sciences applied philosophy to solve real-world wicked problems. Together with translational medicine, ethno-medicine and cross-cultural psychiatry, they pave the way to interdisciplinary dance therapeutic research and its potential to bridge the gap between clinical sciences and ethnocultural traditions.

These challenges require open-minded collaboration that accepts different epistemological and ontological features of medical theories and knowledge alongside mutual appreciation of dance therapeutic traditions, schools and models. This precondition, however, cannot be taken for granted, and we observe several dance therapeutic circles tending to monopolize the ‘market’. By way of illustration, dance/movement therapy DMT (Chaiklin and Wengrower, 2009), which is one of the most popular schools of dance therapy and covers a broad spectrum of clinical applications, e.g., in oncology (Abu-Odah et al., 2024). Despite the wealth of dance therapeutic schools, traditions and models such as dance-rhythm therapy (Schott-Billmann, 2020) or the healing dance Vimbuza of the Tumbuka people living in northern Malawi, which became in 2008 UNESCO Intangible Cultural Heritage of Humanity, numerous people who are involved in arts-based complementary medicine and dance therapy identify dance therapy and dance/movement therapy in a strikingly reductionist way, and the Sri Lankan dance therapy expert Mantillake (2022) claims ‘decolonizing dance movement therapy’. Dance therapeutic research is called to discover and protect the enormous richness of this unlimited realm, and both discussion and action are needed.

## Data Availability

The raw data supporting the conclusions of this article will be made available by the authors, without undue reservation.
